# Application of the combination of three-dimensional visualization with a problem-based learning mode of teaching to spinal surgery teaching

**DOI:** 10.1186/s12909-022-03931-5

**Published:** 2022-12-05

**Authors:** Maji Sun, Fuchao Chu, Chunjiu Gao, Feng Yuan

**Affiliations:** Department of Spine Surgery, The Affiliated Hospital of Xuzhou Medical University, Xuzhou, 221006 Jiangsu People’s Republic of China

**Keywords:** 3D visualization technology, Teaching, PBL

## Abstract

**Objective:**

To explore the application of the combination of three-dimensional visualization technology with a problem-based learning mode of teaching in clinical teaching related to spinal surgery.

**Methods:**

A total of 106 5-year undergraduate students who majored in clinical medicine were selected as research subjects, and practiced in the Orthopaedics Department of the Affiliated Hospital of Xuzhou Medical University in 2021. These students were randomly divided into an experimental group and a control group, with 53 students in each group. The experimental group received a combination of three-dimensional visualization technology with the PBL teaching mode, while the control group was treated with the traditional teaching method. The teaching effects exhibited by these two groups were compared using tests and questionnaires that were administered after the teaching was completed.

**Results:**

The theoretical test total scores of students in the experimental group were higher than those of students in the control group. The students in the two groups self-rated their classroom achievements, and the results attained by students in the experimental group were higher than those obtained by students in the control group (*P* < 0.05). The scores reported by students in the experimental group for interest in learning, classroom atmosphere, classroom interaction and teaching satisfaction were higher than those reported by students in the control group (*P* < 0.05).

**Conclusion:**

The application of a combination of 3D visualization technology with the PBL teaching mode to spinal surgery teaching can improve students’ learning efficiency and interest and is conducive to cultivating students’ clinical thinking.

## Introduction

In recent years, due to the continuous accumulation of clinical knowledge and technology, the question of what kinds of medical education can effectively decrease the time required for the transitional stage from medical student to doctor and lead to the quick cultivation of excellent resident physicians has received a great deal of attention [1]. Clinical practice is an important stage with respect to the cultivation of the clinical thinking and practical ability of medical students. In particular, surgery has strict requirements with respect to students’ hands-on ability and mastery of human anatomy.

At present, traditional lecture-based teaching remains predominant in both schools and clinical medicine [2]. The traditional lecture method is teacher-centred; the teacher stands on the podium and imparts knowledge to the students with the help of conventional teaching methods such as textbooks and multimedia courseware, and the whole course is led by the teacher. Students mainly listen to the lecture and have few opportunities to engage in discussion and ask questions freely. It is thus easy for this process to become a one-way indoctrination by the teacher and for students to accept this situation passively. Therefore, during the teaching process, teachers generally find that students are less motivated to learn, less enthusiastic and less effective. In addition, it is difficult to describe the complex spinal structure clearly using two-dimensional images such as PPT, anatomy textbooks, and pictures, and it is not easy for students to understand and master this knowledge [3].

In 1969, McMaster University School of Medicine in Canada piloted a new teaching method, i.e., problem-based learning (PBL). Unlike traditional teaching methods, the PBL teaching process takes students as the main component of the teaching process and uses relevant questions as clues, thus allowing students to study, discuss and cooperate independently in groups as well as to ask questions and find answers actively, rather than accepting them passively [4, 5]. In the process of analysing and solving problems, students’ autonomous learning ability and logical thinking ability are cultivated [6]. In addition, due to the development of digital medical technology, clinical teaching methods have been greatly enriched. Three-dimensional visualization (3DV) technology allows original data to be obtained through medical imaging, imports modelling software for 3D reconstruction, and subsequently processes the data to produce a three-dimensional model. This method overcomes the limitations of traditional teaching models, mobilizes students’ attention in many ways, and helps students learn complex anatomical structures quickly [7, 8], especially in the context of orthopaedic teaching. Therefore, this paper combines these two approaches to explore the effect of combining PBL with 3DV technology and the traditional teaching mode in practical teaching applications. The results are as follows.

## Materials and methods

### General information

A total of 106 students who entered our hospital as interns in spinal surgery in 2021 were selected as research subjects; these students were divided into an experimental group and a control group using the random number table method, with 53 students in each group. The experimental group included 25 males and 28 females between the ages of 21 and 23 years, with an average age of 22.6 ± 0.8 years. The control group included 26 males and 27 females between the ages of 21 and 24 years, with an average age of 22.6 ± 0.9 years; all students were trainees. There were no significant differences in terms of age or sex between the two groups (*P* > 0.05).

The inclusion criteria were as follows: (1) full-time clinical undergraduate students in their fourth year of study; (2) students who could express their true feelings clearly; and (3) students who could understand and voluntarily participate in the whole process of this study and sign an informed consent form. The exclusion criteria were as follows: (1) students who did not meet any of the inclusion criteria; (2) students who did not want to participate in this training for personal reasons; and (3) students with experience with PBL teaching.

### Methods

#### Preparation of the 3D model

**Fig. 1:**
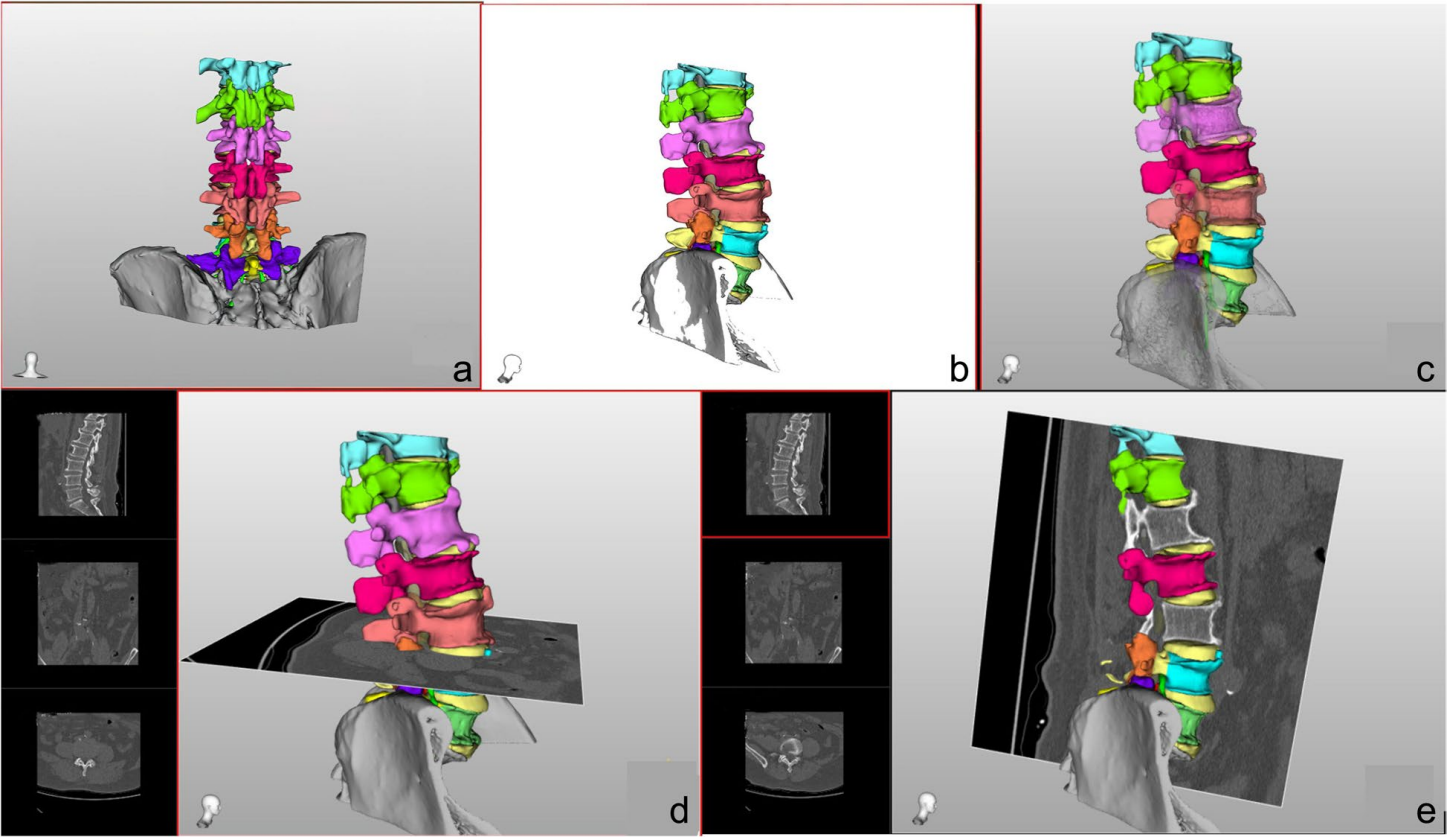
**a** Back view and **b** Side view. **c** The L1, L3 and pelvis of the model were transparent. **d** Combining the CT cross-sectional image with the model, you can slide up and down to adjust different CT planes. **e** CT sagittal image combining the model and processes L1 and L3 with hidden instructions

The original CT data were imported into the modelling software for modelling, and the model thus constructed was imported into the customized teaching software for display. The model consists of bone tissue, intervertebral discs and spinal nerves (Fig. 1). Different parts are represented by different colours, and the model can be magnified and rotated at will. The main advantage of this strategy is that it can locate the CT layer on the model, and the transparency of each part can be adjusted to effectively avoid occlusion.

#### Research methods

The main teaching contents are as follows: 1) the diagnosis and treatment of common diseases in spinal surgery, 2) knowledge of spinal anatomy, consideration and understanding of the occurrence and development of the disease, 3) an operation video intended to teach the basic steps of common spinal surgery, 4) imaging pictures of typical diseases in the context of spinal surgery, 5) classical theoretical knowledge that must be memorized, including Denis’s three-column spine theory, spinal fracture classification, and lumbar disc herniation classification.

Experimental group: A combination of PBL with three-dimensional visualization technology was adopted for teaching. This approach involved the following aspects. 1) The preparation of typical cases of spinal surgery: a discussion of cases of cervical spondylosis, lumbar disc herniation and pyramidal compression fracture was to be created, with each case focusing on different items of knowledge. The case, 3D model and surgery videos were sent to the students 1 week prior to the class, and they were instructed to use the three-dimensional model to review anatomical knowledge. 2) Preclass preparation: 10 minutes prior to class, we introduced the specific process associated with the PBL teaching model to students and encouraged them to participate actively, make full use of their time and complete their tasks reasonably. The groups were divided after obtaining consent from all participants. Sets of 8 to 10 students were free to form groups to consider the case search data, engage in self-study and consideration, participate in group discussions, provide mutual answers, and ultimately summarize the main points, form systematic data, and keep a record of the discussion. A student with strong organizational and expressive skills was selected as the group leader to organize the group discussion and speech. 3) Teacher guidance: teachers used modelling software to explain spinal anatomy by reference to typical cases, and students were allowed to use the software actively to scale, rotate, change CT positioning, set tissue transparency and perform other operations; the purpose of this approach was to allow them to obtain a deeper understanding of and improve their memory regarding the structures surrounding different tissues and to guide them to consider the occurrence and development of the disease and the basic links of the operation independently. 4) Communication and discussion: in response to the questions listed prior to class, class discussion speeches were held, and each group leader was encouraged to report the results of the group discussion after sufficient discussion time. At this time, the groups could ask each other questions and help each other, while the teacher was required to listed carefully and understand the students’ ways of thinking and the associated problems. 5) Summary: After the students’ discussion, the teacher commented on the students’ speeches, summarizes and answers some common and controversial questions in detail, and highlights the direction of future study to allow the students to adapt to the PBL teaching method.

In the control group, the traditional teaching mode was adopted, and the students were instructed to preview the material prior to class. The teachers taught the theory with the assistance of blackboards, multimedia courseware, video materials, specimen models and other teaching aides; the teachers also arranged the teaching progress in accordance with the teaching materials. As a supplement to the syllabus, the focus of this process was on the relevant difficulties and key points in the teaching materials. Following the lecture, the teacher summarized the material to encourage the students to remember and understand the relevant knowledge.

### Comparison of teaching effects

#### Theoretical examination

In the form of closed-paper examinations based on the teaching content, objective questions were selected from the relevant questions offered by medical practitioners over the years, and subjective questions were developed by the orthopaedic teaching and research department and ultimately marked by teachers who did not participate in the teaching. A full score on the examination is 100, and the content mainly includes the following two parts: 1) objective questions (mainly multiple choice questions), which primarily focus on students’ mastery of knowledge items, accounting for 50% of the total score, and 2) subjective questions (case analysis questions), which primarily focus on the students’ systematic mastery and analysis of the disease, accounting for 50% of the total score.

#### Questionnaire investigation

At the conclusion of the course, a questionnaire consisting of two parts featuring a total of nine questions was administered. The main content of these questions corresponded to the items shown in the table, and students were asked to answer the questions based on these items with a full score of 10 and a minimum score of one. Higher scores indicate higher student satisfaction. The items shown in Table 2 focus on whether the combination of PBL with the 3DV teaching model is helpful for students in their attempts to understand complex professional knowledge. The items shown in Table 3 emphasize the students’ satisfaction with the two teaching models.

### Statistical method

All data were analysed using SPSS 25 software; the test scores were expressed as the mean ± standard deviation (x ± s). The quantitative data were analysed conducting a one-way ANOVA, while the qualitative data were analysed using the χ2 test, and multiple comparisons were made using the Bonferroni correction. The difference was significant (*P* < 0.05).

## Results

### Examination results

The results of statistical analysis of the two groups showed that the scores on objective questions (multiple choice questions) achieved by students in the control group were significantly higher than those achieved by students in the experimental group (*P* < 0.05) and that the scores on subjective questions (case analysis questions) achieved by students in the experimental group were significantly higher than those achieved by students in the control group (*P* < 0.01) Table 1.

**Table 1:** The test scores of objective questions and subjective questions were compared between the two groups. ($\overline{x}$± s, points)

Group	Experiment	Contrast	T	*P*
Objective topic	35.94 ± 6.91	39.53 ± 5.77	2.90	0.005
Subjective topic	38.32 ± 5.52	31.40 ± 5.28	6.60	<0.01

### Results of the questionnaire survey

Anonymous questionnaires were distributed after all class hours. A total of 106 questionnaires were distributed, and 106 copies were recovered, for a recovery rate of 100.0%. All questionnaires were completed. Comparison of the results of the questionnaire survey regarding the mastery of professional knowledge between the two groups revealed that the scores of the students in the experimental group were superior to those of students in the control group with regard to learning the basic steps of spinal surgery, the knowledge of planning, the classical classification of diseases and so on. This difference was statistically significant (*P* < 0.05), as shown in Table 2.

**Table 2:** The two groups of students self-rated their professional knowledge. ($\overline{x}$ ± s, points)

Items	Experiment	Contrast	T	*P*
Basic steps of spinal surgery	7.09 ± 1.33	5.77 ± 1.86	4.21	<0.001
Spinal anatomy	6.53 ± 1.85	4.75 ± 2.00	4.75	<0.001
Classic classification of disease	7.72 ± 1.41	7.02 ± 1.54	2.44	0.016
System to study spinal disease	6.26 ± 1.93	5.00 ± 1.95	3.35	0.001
Three-dimensional CT image of reading	6.85 ± 1.71	5.34 ± 1.72	4.53	<0.001

Note: The maximum score for a single item is 10 points

Comparison of the answers to the questionnaires related to teaching satisfaction between the two groups: the scores of students in the experimental group in learning interest, classroom atmosphere, classroom interaction and teaching satisfaction were higher than those of students in the control group. This difference was statistically significant (*P* < 0.05). The details are shown in Table 3.

**Table 3:** Comparison of comprehensive evaluation and teaching satisfaction between the two groups of students

Items	Experiment	Contrast	T	*P*
Interest in learning	7.30 ± 1.14	5.66 ± 1.70	5.85	<0.001
Classroom atmosphere	6.98 ± 1.23	5.75 ± 1.57	4.48	<0.001
Classroom interaction	7.13 ± 1.30	5.32 ± 1.25	7.30	<0.001
Teaching satisfaction	8.17 ± 1.46	7.32 ± 1.76	2.70	0.008

## Discussion

Due to the continuous accumulation and development of science and technology, especially since the beginning of the twenty-first century, clinical work in hospitals has become increasingly complex. To ensure that medical students can adapt to clinical work quickly and train high-quality medical talent for the benefit of society, the traditional instillation and unitary teaching model faces difficulties with respect to solving clinical practical problems. The traditional model of medical education in China offers the advantages of a large amount of classroom information, low environmental requirements, systematic teaching knowledge and so on, which can basically meet the teaching needs of theoretical courses [9]. However, this teaching model can easily lead to a disconnection between theory and practice, decrease students’ learning initiative and enthusiasm, and fail to analyse complex diseases in clinical practice comprehensively; accordingly, it cannot meet the requirements of higher medical education. In recent years, rates of spinal surgery in China have increased rapidly, and the teaching of spinal surgery has ushered in new challenges. During the undergraduate study of medical students, the most difficult aspect of surgery is orthopaedics, especially spinal surgery, whose knowledge points are relatively trivial, involving not only spinal deformity and infection but also involve trauma, bone tumours, and so on. These concepts are not merely abstract and complex but are also closely related to anatomy, pathology, imaging, biomechanics and other disciplines, so these contents are difficult to understand and remember. Simultaneously, many fields of spinal surgery are developing rapidly, and the knowledge contained in the available teaching materials is out of date, which makes it more difficult for teachers to teach. Therefore, transitioning away from traditional teaching methods in conjunction with the latest progress in international research, can allow the teaching of relevant theoretical knowledge to be connected to practice, improve students’ logical thinking ability, and induce students to engage in critical thinking. These failures of the current teaching process must urgently be addressed to explore the boundaries and limitations of contemporary medical knowledge and overcome the traditional barriers [10].

The PBL teaching mode is a learner-centred teaching method. Through the use of heuristics, independent learning and interactive discussions, students become fully motivated to transition from the passive acceptance of knowledge to active participation in the teacher’s lessons. Compared with the lecture-based teaching model, students who participate in the PBL teaching mode have sufficient time to use textbooks, the internet and software to find answers to their questions, think independently and discuss relevant topics in group settings. This approach develops students’ ability to think about problems, analyse them and solve them independently [11]. In the process of free discussion, different students may have many different understandings of the same problem, which provides a platform for students to expand their thinking. Creative thinking and logical reasoning skills are developed through continuous thinking, and oral expression skills and team spirit are developed through communication among classmates [12]. Most importantly, PBL teaching enables students to understand how to analyse, organize and apply the relevant knowledge, master the correct learning methods, and improve their comprehensive ability [13]. Throughout our teaching process, we found that students were more interested in learning how to use the 3D visualization software than in understanding the boring professional medical concepts covered by textbooks, so in our study, the students in the experimental group were generally more motivated to engage in the learning process than the control group. Teachers should encourage students to speak boldly, cultivate students’ subject consciousness and stimulate their interest in participating in discussions. The test results show that with regard to the knowledge of mechanical memory, the scores of students in the experimental group were lower than those of students in the control group; however, with respect to the clinical case analysis questions that require the comprehensive use of relevant knowledge, the scores of students in the experimental group were significantly better than those of students in the control group, thus highlighting the advantages of the combination of 3DV with the PBL teaching method with regard to cultivating students’ comprehensive ability.

Anatomy teaching is the focus of the clinical teaching associated with spinal surgery. Because the structure of the spine is complex and because this surgery is involved to the spinal cord, spinal nerve, blood vessels and other important tissues, students’ spatial imagination is required for them to learn. Previous trainee teaching has used textbook illustrations, imaging images and other two-dimensional pictures to explain the relevant know, but despite these many materials, students do not develop intuitive, three-dimensional feelings in this context, resulting in difficulties in understanding. In view of the more complex physiological and pathological characteristics of the spine, such as the relationship between the spinal nerve and segments of the vertebral body, the teaching effect is not as strong as expected with regard to certain important and difficult points, such as the characteristics and classification of cervical fracture. Many students report that the content of spinal surgery is abstract and cannot be fully understood when learning, and the knowledge that they do remember will be forgotten soon after class, leading to difficulties in actual operations.

The author uses three-dimensional visualization technology to present clear and three-dimensional images to students, with different parts being represented by different colours. Through rotation, scaling, transparency and other operations, the spinal model can be viewed in layers alongside CT images, which not only clearly allows the students to observe the anatomical features of the cone but also stimulates their desire to engage in otherwise boring spinal CT imaging learning and deeply strengthens their imaging knowledge. Unlike the teaching models and tools used previously, the transparent processing function can effectively solve the occlusion problem, and it is more convenient by allowing students to observe fine anatomical structures and complex nerve directions, especially for beginners. Students can operate freely as long as they bring their own computers, and there are virtually no associated costs. This approach is a perfect substitute for traditional two-dimensional image teaching [14]. In our study, the control group performed better with regard to objective questions, indicating that the lecture teaching model cannot be rejected entirely, since it still has a certain degree of value in the clinical teaching of spinal surgery. This finding leads us to consider whether we should combine the traditional teaching model with the PBL teaching model as supplemented with 3D visualization technology for different types of tests and different levels of students with the aim of maximizing educational outcomes. However, whether and how the two can be combined and whether students are receptive to this combination remains unclear, which may be a direction for future research. This study also faces certain shortcomings, such as possible confirmation bias in students’ completion of the questionnaires after they became aware that they would be participating in a new educational model. This teaching experiment was implemented only in the context of spinal surgery and requires further validation if it is to be applied to the teaching of all surgical disciplines.

We combine 3D visualization technology with the PBL teaching model to overcome the limitations of traditional teaching models and teaching tools and to explore the practical application of this combination to the clinical probation teaching of spinal surgery. With regard to the examination scores, the scores of students in the experimental group on subjective questions were better than those of students in the control group (*P* < 0.05), and the professional knowledge and classroom satisfaction reported by students in the experimental group in the questionnaire survey were better than those reported by students in the control group (*P* < 0.05). In summary, our experiments confirmed that the combination of PBL with 3DV technology is beneficial and allows students to engage in clinical thinking, learn professional knowledge and improve their interest in learning.

## Conclusion

A combination of PBL with 3DV technology can effectively improve the clinical trainee performance of medical students in the context of spinal surgery, increase students’ learning efficiency and interest, and help cultivate students’ clinical thinking. Three-dimensional visualization technology offers significant advantages in the context of anatomy teaching, and the overall teaching effect associated with this approach is superior to that of the traditional teaching mode.

## Data Availability

The datasets used and/or analyzed during the current study are available from the corresponding author on reasonable request. We do not have ethical permission to upload the dataset into a repository. Please note that all study data has been anonymised for confidentiality purposes.
